# Economic benefits of safety-engineered sharp devices in Belgium - a budget impact model

**DOI:** 10.1186/1472-6963-13-489

**Published:** 2013-11-25

**Authors:** Emma Hanmore, Grant Maclaine, Fiona Garin, Alexander Alonso, Nicolas Leroy, Lewis Ruff

**Affiliations:** 1 Medaxial Ltd., London, UK; 2 Becton, Dickinson UK Ltd., Oxford, UK; 3 Becton, Dickinson SA, Madrid, Spain; 4 Becton, Dickinson Benelux NV, Erembodegem, Belgium

**Keywords:** Budget, Model, Economic, Needlestick, Safety-engineered, Device

## Abstract

**Background:**

Measures to protect healthcare workers where there is risk of injury or infection from medical sharps became mandatory in the European Union (EU) from May 2013. Our research objective was to estimate the net budget impact of introducing safety-engineered devices (SEDs) for prevention of needlestick injuries (NSIs) in a Belgian hospital.

**Methods:**

A 5-year incidence-based budget impact model was developed from the hospital inpatient perspective, comparing costs and outcomes with SEDs and prior-used conventional (non-safety) devices. The model accounts for device acquisition costs and costs of NSI management in 4 areas of application where SEDs are currently used: blood collection, infusion, injection and diabetes insulin administration. Model input data were sourced from the Institut National d’Assurance Maladie-Invalidité, published studies, clinical guidelines and market research. Costs are discounted at 3%.

**Results:**

For a 420-bed hospital, 100% substitution of conventional devices by SEDs is estimated to decrease the cumulative 5-year incidence of NSIs from 310 to 75, and those associated with exposure to blood-borne viral diseases from 60 to 15. Cost savings from managing fewer NSIs more than offset increased device acquisition costs, yielding estimated 5-year overall savings of €51,710. The direction of these results is robust to a range of sensitivity and model scenario analyses. The model was most sensitive to variation in the acquisition costs of SEDs, rates of NSI associated with conventional devices, and the acquisition costs of conventional devices.

**Conclusions:**

NSIs are a significant potential risk with the use of sharp devices. The incidence of NSIs and the costs associated with their management can be reduced through the adoption of safer work practices, including investment in SEDs. For a Belgian hospital, the budget impact model reports that the incremental acquisition costs of SEDs are offset by the savings from fewer NSIs. The availability of more robust data for NSI reduction rates, and broadening the scope of the model to include ancillary measures for hospital conversion to SED usage, outpatient and paramedic device use, and transmission of other blood-borne diseases, would strengthen the model.

## Background

### The burden of needlestick injuries

Needlestick injuries (NSIs) are one of the most common and serious risks to European healthcare workers. Such injuries occur before, during and after sharp device use, and affect both direct patient care staff and ancillary staff, such as sanitation workers [1]. Data from the United States (US) EPINet registry show that 34% of NSIs reported to it are experienced by staff other than the user of the sharp [2].

Over 1 million NSIs are estimated to occur in the European Union (EU) each year [3] and true injury rates may be far higher due to under-reporting, which is estimated to occur in 29–61% of cases [4]. Reasons for under-reporting include: a presumption that the risk of disease transmission is low; a lack of knowledge of systems for, or the necessity of, reporting NSIs; and reporting systems that are cumbersome or unclear.

The main risk following an NSI is infection with a blood-borne virus [5]. This risk is exacerbated by the heightened prevalence of these viral infections in hospital patients compared to the general population – significantly so in the case of hepatitis B, hepatitis C and human immunodeficiency virus (HIV), as demonstrated by Wicker *et al*. [6].

NSIs represent a significant clinical and economic burden to healthcare systems and society [7]. The economic burden of NSIs facing healthcare employers and insurers was shown in a costing statement published by NHS Scotland [8] to stem from costs such as:

•Testing for infection in the injured worker and, if known, the patient on whom the sharp had been used.

•Post-exposure prophylaxis (PEP) to prevent or manage potential blood-borne virus transmission.

•Short- and long-term treatment of chronic blood-borne viral infections that are transmitted to injured workers.

•Staff absence and replacement.

•Counselling for injured workers.

•Legal consequences (e.g. litigation and compensation claims).

Estimates of the economic burden of NSIs are considerable but vary across countries and studies due to the different study methodologies used. For example, Saia *et al*. reported annual costs due to NSIs of between €4.6 million and €30 million in Germany, $6.1 million in France (considering nurses alone), €7 million in Italy (not considering long-term treatment, compensation or indirect costs), between €6 million and €7 million in Spain, and between £4 million and £300 million in England and Wales [9].

NSIs may also have a detrimental impact on healthcare workers’ psychological well-being or quality of life. For instance, in one survey of workers exposed to blood or body fluids, 53% reported symptoms of anxiety and 18% symptoms of depression [10]. Many respondents reported that they were worried for up to a year after the incident even when the source patient was confirmed negative of infection. In a Korean survey conducted by Sohn *et al*. [11], 370 healthcare workers’ self-reported levels of anxiety and depression were elevated after becoming injured and stress levels increased in those who were not vaccinated against hepatitis B.

In a US survey of 400 acute care nurses [12], 110 had experienced an NSI in the previous 12 months. Of these, 42% reported that they were anxious, depressed or stressed during the two-week period following injury and 60% reported feeling more afraid of needled devices than before. In the 12-month period, these NSIs generated 19 counselling visits and 61 days of work absence due to emotional distress and anxiety alone. The same study reported that an additional 10 days of work were missed as a result of seeking and receiving medical attention.

Fear of transmission of chronic blood-borne virus infection is particularly impactful on a healthcare worker’s well-being. In a survey of accident and emergency doctors, over 70% indicated that their worst fear was an NSI associated with care of an HIV-infected patient [13]. In another study among doctors, focused specifically on the impact of HIV infection risk on outlook and behaviour, most stress was reported by those who had previously experienced an NSI [13].

### The European directive

In recognition of the burden of sharps injuries including NSIs, an EU Directive on the prevention of sharps injuries (Council Directive 2010/32/EU) was adopted into European law in May 2010 [14]. The Directive calls for the elimination of risk to the maximum degree possible and, beginning with mandatory risk assessment, it recommends adherence to a range of measures to eliminate risk where it exists to prevent injury and protect healthcare workers, including:

•Implementing safe procedures for using and disposing of sharps and contaminated waste.

•Eliminating unnecessary use of sharps, including provision of devices with safety-engineered protection mechanisms.

•Implementing safe systems of work including training and health surveillance procedures.

•Provision of personal protective equipment.

•Vaccination.

The deadline for compliance to the directive for EU member states was 11^th^ May 2013.

### Safety-engineered devices (SEDs)

SEDs are engineered with automatic or manually-activated shields or retraction mechanisms designed to protect the device user from exposure to the sharp by covering the sharp immediately following use. Typically, the safety mechanism is either automatic or engineered for rapid and intuitive activation, which for some devices is single-handed for convenience and ease of use.

A French, multicentre, prospective survey conducted across 102 medical units to explore the effect of the introduction of SEDs when conducting sharp procedures found that, in combination with training and safer working practices, there was a 75% reduction in the incidence of NSIs [15].

The addition of safety features increases the acquisition cost of these devices relative to conventional devices. Accordingly, we developed a budget impact model to understand the potential overall economic impact of introducing SEDs in Belgian hospitals.

## Methods

### Model design

An incidence-based, deterministic budget impact model with a five-year time horizon was developed from the perspective of a Belgian hospital, focusing on the inpatient setting. Pertinent direct and indirect costs were considered.

The model was developed in accordance with recommendations published by the International Society for Pharmacoeconomics and Outcomes Research (ISPOR) Task Force on Good Research Practices [16], as far as possible. It takes into account device acquisition costs and other direct and indirect costs associated with NSIs in four clinical applications where SEDs are currently in use:

•Injection.

•Infusion therapy.

•Blood collection.

•Diabetes insulin administration.

Current and revised care pathways were compared. In the base case scenario it was assumed that no SEDs are used in the current care pathway and that SED use is 100% in the revised care pathway. All model inputs were sourced from a targeted literature review. Belgian data sources were used where possible for model input data; otherwise the next most suitable sources were used.

### Parameters

#### Devices and incidence of NSIs

The average number of relevant devices used in a Belgian hospital was based on an estimate of the average number of beds per hospital – 420 [17] – and the annual number of devices used per bed, based on market research data from Becton, Dickinson and Company, in addition to procurement data for blood collection devices used per inpatient bed reported by NHS Scotland [8] (it was assumed that each device is a single-use device and not reused). NSI rates associated with use of conventional devices and the expected reduction in NSI rates associated with SEDs were based on Lamontagne *et al*. [15] and Pellisier *et al*. [18]. These data are shown in Table 1.

**Table 1:** Estimated annual number, cost and NSI rate associated with procedures in Belgium

**Procedure class**	**Annual number of devices per bed (estimated)**	**Average cost of conventional devices (€)**	**Average cost of SEDs (€)**	**NSI rate using conventional devices(per 10**^**5**^**procedures)**	**NSI rate using SEDs (per 10**^**5 **^**procedures)**[15]
Injection	553	0.014	0.046	10.8 [15]	1.5
Infusion therapy	120	0.400	0.600	26.0 [15]	8.8
Diabetes insulin administration	50 [8]	0.095	0.315	23.5 [18]	3.3**
Blood collection	193	0.170	0.270	23.4* [15]	7.0***

*It was assumed that arterial and venous blood collection procedures result in the same percentage of NSIs.

**It was assumed that the reduction in NSIs after the introduction of 2^nd^ generation safety-engineered diabetes care devices is the same as that achieved by injection SEDs.

***It was assumed that safety-engineered arterial and venous blood collection devices achieve the same reduction.

#### Transmission of blood-borne viruses

The risk of contracting hepatitis B, hepatitis C and HIV from an NSI was calculated based on:

•The prevalence of these diseases in a hospital population (shown in Table 2), derived from a study of reported exposures to blood between 2003 and 2010 in a major academic medical centre [19].

•A disease transmission rate of 30%, 1.8% and 0.31%, respectively [20,21], (shown in Table 2).

•The proportion of healthcare workers vaccinated against hepatitis B – 89%, based on a published European range of 85–93% reported by De Schryver *et al*. [22].

•Prior effectiveness of hepatitis B vaccination deemed to be 90%, based on a reported range of 85–95% effectiveness in preventing hepatitis B infection [23].

**Table 2:** Estimated prevalence and transmission rates of blood-borne viruses

	**Hepatitis B**	**Hepatitis C**	**HIV**
Prevalence in a hospital population [19]	4.9%	7.9%	9.0%
Transmission rate	30% [20]	1.8% [20,21]	0.31% [20,21]

The model also factors in the percentage of cases where the source of an NSI is unknown (23%) [24]. Cases such as these, as well as those cases where the source patient is known to carry one or more of the three considered blood-borne viral infections, are treated as high-risk NSIs and make up 39% of the NSIs that occur. Low-risk NSIs – those where the source patient is identified and confirmed as uninfected with HIV, hepatitis B or hepatitis C – make up the remaining 61%.

#### Costs

The direct and indirect costs of managing an NSI were assumed to be the same across all clinical applications. It was assumed that the use of SEDs only affects the overall number of NSIs. The proportions of high- and low-risk NSIs were assumed to be the same with the usage of both SEDs and conventional devices.

Belgian unit costs were taken from the Institut National d’Assurance Maladie-Invalidité (INAMI) database [25,26], where possible.

The direct cost parameters included in the model are outlined in Table 3.

**Table 3:** Direct costs associated with NSIs

**Direct costs of NSIs**	
Testing for blood-borne viruses [6,26-30] in:	
*NSI recipient – initial test*	€38.76
*NSI recipient – follow-up test*	€58.52
*Source patient (if known)*	€43.91
Cost of staffing and administration per NSI [26,31]	€68.81
Cost of PEP for hepatitis B and HIV [19,25,32,33] per:	
*NSI from a known and infected source*	€374.61
*NSI from an unknown source*	€918.76
Cost of treatment of blood-borne viruses [19,25,34-38]:	€2,377.44
*Hepatitis B (year in which NSI occurred and per subsequent year for three years)*	
*Hepatitis C (year in which NSI occurred only)*	€16,127.76
*HIV (year in which NSI occurred and per subsequent year)*	€16,999.83

Costs associated with testing for blood-borne viral infections after an NSI were based on a regimen determined from clinical guidelines [27-29], peer-reviewed studies [6,30] and INAMI costs [26]. The costs of staff time required for administrative procedures following an NSI were based on resource use estimates from a Belgian macroeconomic study [31] and INAMI unit costs [26]. It was assumed that workers do not resign after NSIs, so the model does not factor in costs associated with personnel replacement.

Based on Belgian clinical guidelines [32,33], PEP for hepatitis B and HIV was applied to victims of NSIs arising from a known and infected source or an unknown source. Hepatitis B PEP consisted of a course of four doses of hepatitis B recombinant vaccine for unvaccinated workers and one dose for vaccinated workers, whilst HIV PEP consisted of 28 days treatment with a combination of lopinavir, ritonavir, emtricitabine and tenofovir disoproxil. It was assumed that where the source patient is known and infected, the average cost of PEP with HIV and hepatitis B would be applied, weighted by the prevalence of these blood-borne viruses [19], and where the source patient is unknown the cost of both hepatitis B and HIV PEP would also be applied.

Patients treated for:

•Hepatitis B receive either peginterferon, lamivudine, adefovir dipivoxil or entecavir, as documented by the United Kingdom (UK) National Institute for Health and Care Excellence (NICE) [34].

•Hepatitis C receive combination therapy with peginterferon alfa and ribavirin [35] for 48 weeks, according to NICE guidance [36].

•HIV incur an annual treatment cost based on the annual cost of antiretroviral therapy reported in the UK for 2006 [37], converted to 2012 Euro [38], making the conservative assumption that all patients remain asymptomatic in the first five years following infection and receive monotherapy treatment.

INAMI drug costs were used for both PEP and treatment of blood-borne viruses [25].

The overall direct cost per NSI is shown in Table 4.

**Table 4:** Average direct costs incurred per low- and high-risk NSI

**NSI risk category**	**Low**		**High**	
Source patient	Known		Unknown	
Infection status	Uninfected	Infected	Uninfected	Infected
Testing	€141.20	€141.20	€85.09	€85.09
Staff and administration	€68.81	€68.81	€68.81	€68.81
PEP	–	€374.61	€918.76	€918.76
Disease treatment	–	€142.99	–	€142.99
Total direct cost per NSI	**€210.01**	**€727.61**	**€1,072.66**	**€1,215.65**
Relative incidence within risk category	**100%**	**33%**	**58%**	**10%**
Total direct cost per NSI, by risk category	**€210.01**	**€950.34**		

The model includes indirect costs of NSIs, namely: counselling NSI victims, staff absence and compensation. It was assumed that all victims of NSIs would require at least an initial counselling session, with recipients of high-risk NSIs requiring a further three follow-up sessions [39], costed using INAMI unit cost data [26]. The cost of staff absences was based on a report by the Netherlands National Hepatitis Centre [40], and productivity loss costs were derived from Belgian national statistics [41]. It was conservatively assumed that no low-risk NSIs result in compensation claims. For high-risk NSIs, it was assumed that 4% result in compensation [12]. The estimated compensation cost per NSI was based on UK experience [42-44] converted to 2012 Euro using historical exchange rates [38].

Average indirect costs incurred per NSI are shown in Table 5.

**Table 5:** Average indirect costs incurred per low- and high-risk NSI

**Risk category**	**Low**	**High**
Counselling [26,39]	€40.27	€238.45
Staff absence [40,41]	€22.95	€229.51
Compensation and litigation [12,38,42-44]	–	€376.26*
Total indirect cost per NSI	**€63.22**	**€844.22**

*Estimated based on an average of the costs reported by three English compensation cases involving a healthcare worker experiencing an NSI [42-44], weighted by the relative proportion of high-risk NSIs resulting in seroconversion to either hepatitis B, hepatitis C or HIV from an infected source, and converted to 2012 Euro [38].

Costs were discounted at 3%, in line with Belgian guidelines for pharmacoeconomic evaluation [45]. The model conservatively assumes that only costs (device costs and direct and indirect costs relating to NSIs) incurred within the model time horizon are included. This may understate the true cost of chronic blood-borne diseases, such as those for HIV infection, where ongoing lifetime treatment is required.

### Sensitivity analysis

One-way sensitivity analyses were performed by varying the following model parameters:

•Number of hospital beds (±20%).

•Number of devices used (±20%).

•Cost per conventional device (±20%).

•Cost per SED (±20%).

•Rate of NSIs with conventional devices (±20%).

•Reduction in NSIs with SEDs (±20%).

•Prevalence of hepatitis B, hepatitis C and HIV (±20%).

•Rate of transmission of hepatitis B, hepatitis C and HIV (±20%).

•Cost of HIV treatment – the upper and lower bounds (€16,999.83 and €40,336.42) corresponding to the lowest and highest estimated annual costs of retroviral therapy for asymptomatic patients in the UK for 2006 [37], converted to 2012 Euro [38].

•The proportion of workers vaccinated against hepatitis B set to 85% and 93% – the upper and lower bounds reported by De Schryver *et al.*[22].

•Direct cost per NSI (±20%).

•Indirect cost per NSI (±20%).

In addition, the following scenarios were modelled:

•All compensation and litigation costs excluded from the model.

•Uptake of SEDs decreased to 75% and 50% of all devices used.

## Results

### Base case scenario

We estimated that a 420-bed hospital will conduct 384,720 sharps procedures per annum. Over five years, the modelled cost of conducting these procedures using conventional devices was estimated to be €443,120, of which €258,270 (58%) was spent managing 310 NSIs.

**Figure 1:**
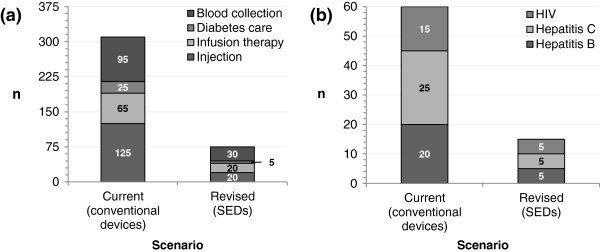
**Estimated incidence of events over the model’s five-year time horizon. (a)** Number of NSIs experienced, and **(b)** total healthcare worker exposures to blood-borne diseases.

**Figure 2:**
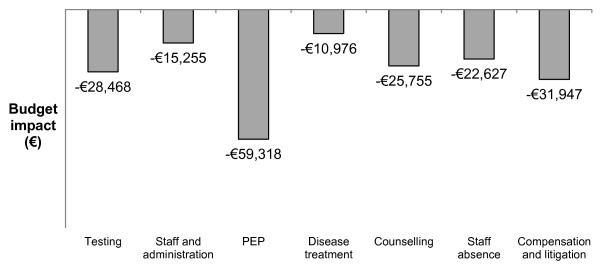
Breakdown of NSI management cost savings, by cost category.

If all procedures were instead conducted using SEDs, the model estimated that the number of NSIs would be reduced to 75, avoiding 235 NSIs and 45 exposures to blood-borne viruses, as shown in Figure 1. Total costs with SEDs were estimated to be €391,450, representing an overall cost saving of €51,710 or 12%. A €142,640 increase in device acquisition costs is offset by a €194,350 reduction in NSI management costs. These overall cost savings are primarily attributable to a reduction in the cost of PEP, as shown in Figure 2.

A threshold analysis estimated that SEDs would still confer cost savings if the average NSI rate reduction was as low as 32% across the four clinical applications.

### Sensitivity analysis results

**Figure 3:**
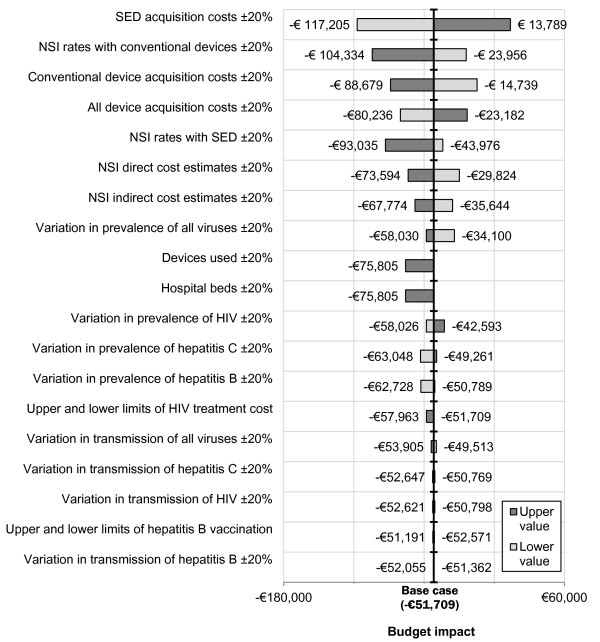
Results of one-way sensitivity analyses.

The results of the one-way sensitivity analyses are shown in Figure 3. The model was most sensitive to variation in the acquisition costs and rates of NSIs associated with both SEDs and conventional devices. For changes in the acquisition costs of SEDs, the range between the upper and lower bounds of net hospital budget impact was €103,420. The model was relatively insensitive to changes in variables related to blood-borne viruses.

**Figure 4:**
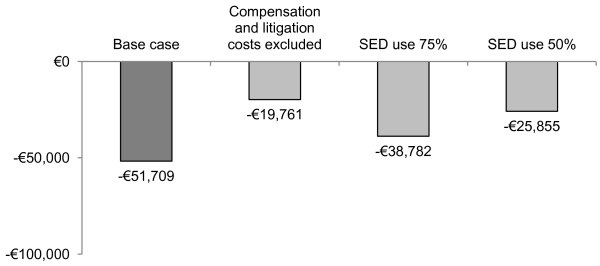
Results of scenario analyses.

The results of scenario analyses are presented in Figure 4. Reducing the uptake of SEDs resulted in lower overall cost savings. The exclusion of compensation and litigation costs had a larger effect, with overall cost savings being reduced to €19,760.

## Discussion

The model demonstrates that the healthcare costs associated with NSIs to hospital workers can be substantial. NSI costs can be reduced and healthcare workers protected by investment in SEDs, the acquisition costs of which may be offset by the savings from fewer NSIs.

The model suggests that a hospital with 420 beds would expect to see a reduction of over 70% in the number of NSIs it would face when using conventional sharp devices, from an estimated 310 NSIs over five years to 75 NSIs following introduction of SEDs. This would lead to 45 fewer exposures (over five years) to HIV, hepatitis B and hepatitis C viruses. The model predicts an annual NSI rate of 16.1 per 100,000 procedures with conventional devices and 3.9 per 100,000 procedures with SEDs. These rates are similar to those observed in a French study conducted in the 1990–7 period which observed pre-conversion NSI rates of 12.7 per 100,000 needles compared to 6.4 per 100,000 needles post-conversion [46]. However, comparison is not straightforward. The period covered by this study suggests an older generation of SEDs with different features and potential for reductions in rates of NSI. No information is given as to whether conversion to SEDs was 100% and there is no information on the comprehensiveness of reporting of NSIs. Regarding the latter, the study may be vulnerable to under-reporting in 1990 during the period of conventional device usage, and much fuller reporting in 1995–7 with other policy changes introduced concurrent to the introduction of SEDs: healthcare worker education and a change in French worker compensation policy.

In May 2013, measures to prevent injuries, where there is a risk of injury or infection from a medical sharp, were legally mandated by the EU Directive [14]. In addition to the primary purpose of improving healthcare worker safety, the current model suggests that investment in safety devices carries economic benefits. Extrapolating the estimated net cost savings of approximately €51,700 per 420-bed hospital over five years, and assuming similar savings across all 141 general hospitals in Belgium [17], the model results suggest an overall national budget saving of approximately €7,300,000 could be achieved over five years. This estimate is broad and does not take account of national variation in hospital size, variation in usage of sharps devices across hospitals or variation in the existing usage of SEDs. The market place for sharps devices is also competitive with device unit acquisition costs varying by time and place, so budget impact analyses are both institution- and time-specific.

Sensitivity analyses show that for all of the varied parameters, with the exception of acquisition costs for SEDs, the model estimates overall hospital budget cost savings. However, for some parameters, such as the acquisition costs for SEDs, rates of NSI associated with conventional devices and the acquisition costs of conventional devices, there is a wide range of uncertainty about the magnitude of overall hospital budget impact.

Other analyses from the published literature also report economic benefits from the implementation of SEDs. A UK cost-benefit analysis of two alternative European Commission strategies (legislative vs. non-legislative) to protect healthcare workers, including the use of SEDs, estimated annual net benefits of £1 million to £5 million over 10 years in 2008 GBP [47]. A Swedish analysis, using clinical and economic data from a sample of 18 hospitals, estimated savings of €850,000 in 2007 Euro from introducing safety needles and syringes in all Swedish hospitals [48]. However, the analysis excluded productivity costs and costs of chronic infection acquired from NSIs, and no account was made of any additional costs for the purchase of SEDs.

Studies conducted in the US have shown similar trends: in 2000, the US General Accounting Office (GAO) concluded that switching to SEDs could avoid 139,000 NSIs and save up to $90 million over one year, depending on the cost of SEDs relative to conventional devices and the cost of post-exposure treatment [49]. NSIs also generate significant intangible costs in the form of the anxiety and distress of injured healthcare workers and their families. A survey of 116 recently-injured healthcare workers found that they would be willing to pay $850 to avoid an NSI – an amount which increased to $1,270 when higher risk was factored in (e.g. working with a difficult patient or a patient infected with hepatitis B, hepatitis C or HIV) – and is within the range of the direct cost of $500–$2,500 per NSI as approximated by the US GAO [49,50].

Although not directly comparable to the budget impact analysis approach used in our study, European cost-effectiveness analyses of introducing measures to prevent NSIs have also been undertaken. In their study of a 1,300-bed hospital in Spain, Armadans Gil *et al.*[51] found that safety needles for implanted ports were cost-effective, despite not including important costs such as compensation and counselling: the cost effectiveness of SEDs ranged from savings of €2.65 (for implanted ports) to costs of €13,564 (for short catheters) per NSI avoided, in 2003 Euro. Roudot-Thoraval *et al*. [46] reported that for a 1,050-bed hospital in France, a combined intervention of healthcare worker education and conversion to SEDs was associated with a 75% and 50% reduction in re-capping rates and NSIs over one year, respectively, at a cost of $4,000 per injury avoided.

One area in which the model is limited is that survey data are used to estimate the reduction in NSIs arising from the use of SEDs [15,18], and underreporting of the true number of NSIs may underestimate the true reduction in NSIs expected with these devices. This underreporting is likely to result in an underestimation of the cost savings associated with switching from conventional devices to SEDs, since fewer than the true number of healthcare workers expected to incur direct and indirect costs associated with NSI management will be modelled. The scale of this issue is highlighted by evidence that between 29–61% of healthcare workers who experience an NSI do not report it [52]. The model does not explicitly consider the cost of other ancillary measures to convert a hospital from conventional to SED usage – such measures might include risk assessment, staff training, work practice redesign and controls implementing safer usage and handling procedures, provision of personal protective equipment and routine worker vaccination against blood-borne diseases.

A further limitation of the model is that outpatient and paramedic device use are not considered. Their inclusion would be expected to result in an increase in both the incidence of NSIs, and therein the number of NSIs avoided through conversion from conventional devices to SEDs, thereby increasing both the potential clinical and economic benefits estimated by the model. In addition, infections from around 30 pathogens transmittable through NSIs [3], beyond the three blood-borne viruses considered, are not included in the model. Again, the model is conservative in this limitation: an expansion of the transmissible infections considered would widen the scope of estimated benefits from the use of SEDs.

The robustness of the results could be improved if better data sources were available for the model input variables. For example, observational study data on NSI rates with conventional devices compared with SEDs would be preferable to survey data. Similarly, the use of Belgium-specific data would be preferable to using data from other Northern European countries where Belgian data are currently lacking, such as for the prevalence of blood-borne viral diseases in hospital patients and compensation costs.

Our study provides a current analysis: many published studies of this nature are now relatively old; in a recently-conducted systematic review [53] two thirds of identified studies were carried out before 2001. Our analysis is also comprehensive with respect to inclusion of important pertinent costs and undertakes extensive sensitivity and scenario analyses. In the existing published literature, some relevant costs are often not taken into account and uncertainty analysis is often lacking.

## Conclusion

Costs associated with NSIs are a significant and avoidable cost of using sharps devices. This model suggests that SEDs reduce the economic burden of managing NSIs, which may partially or completely offset any increase in device acquisition costs. Associated health and quality of life benefits for healthcare workers from a reduction in rates of NSI can be achieved alongside potential reductions in Belgian health system expenditures through conversion to using safety-engineered sharps devices.

## Competing interests

EH was an employee of Medaxial Limited during her participation in the study. LR is an employee of Medaxial Limited. Medaxial Limited received funding from Becton, Dickinson and Company to conduct this study and draft the manuscript. GM, FG, AA and NL are employees of Becton, Dickinson and Company.

## Authors’ contributions

EH participated in the design of the study, developed the model, conducted the base case analyses and critically reviewed the manuscript. LR participated in the design of the study, drafted the manuscript and critically reviewed the manuscript. GM and FG conceived and participated in the design of the study, assisted in the development of the model and critically reviewed the manuscript. AA and NL assisted with the acquisition of data for use in the model and critically reviewed the manuscript. All authors have read and approved the final manuscript.

## Pre-publication history

The pre-publication history for this paper can be accessed here:

http://www.biomedcentral.com/1472-6963/13/489/prepub
